# Direct observation of aggregate-triggered selective autophagy in human cells

**DOI:** 10.1242/jcs.258824

**Published:** 2021-10-06

**Authors:** Anne F. J. Janssen, Giel Korsten, Wilco Nijenhuis, Eugene A. Katrukha, Lukas C. Kapitein

**Affiliations:** 1 Cell Biology, Neurobiology and Biophysics, Department of Biology, Faculty of Science, Utrecht University, Padualaan 8, 3584 CH Utrecht, The Netherlands; 2 Cambridge Institute for Medical Research, University of Cambridge, Cambridge Biomedical Campus, Hills Road, CB2 0XY Cambridge, UK; 3 Center for Living Technologies, Eindhoven-Wageningen-Utrecht Alliance, Padualaan 8, 3583 CH Utrecht, The Netherlands

**Keywords:** Autophagy, Aggregates, Live-cell imaging

## Abstract

Degradation of aggregates by selective autophagy is important as damaged proteins may impose a threat to cellular homeostasis. Although the core components of the autophagy machinery are well characterized, the spatiotemporal regulation of many selective autophagy processes, including aggrephagy, remains largely unexplored. Furthermore, because most live-cell imaging studies have so far focused on starvation-induced autophagy, little is known about the dynamics of aggrephagy. Here, we describe the development and application of the mKeima-PIM assay, which enables live-cell observation of autophagic turnover and degradation of inducible protein aggregates in conjunction with key autophagy players. This allowed us to quantify the relative timing and duration of different steps of aggrephagy in human cells and revealed the short-lived nature of the autophagosome. The assay furthermore showed the spatial distribution of omegasome formation, highlighting that autophagy initiation is directly instructed by the cargo. Moreover, we found that nascent autophagosomes mostly remain immobile until acidification occurs. Thus, our assay provides new insights into the spatiotemporal regulation and dynamics of aggrephagy.

This article has an associated First Person interview with the first author of the paper.

## Abstract (precis)

**Summary:** We describe an assay that enables live-cell imaging of the autophagic turnover and degradation of inducible protein aggregates in conjugation with key players in the autophagy pathway.

## INTRODUCTION

Efficient turnover of misfolded proteins and damaged or redundant organelles is essential to maintain cellular homeostasis, and cells have evolved multiple pathways to ensure (protein) quality control. Misfolded proteins can be refolded by chaperone networks or degraded via the ubiquitin-proteasome system or the autophagy-lysosome pathway. Superfluous organelles can be degraded as a whole by macroautophagy (autophagy hereafter). Although autophagy was initially characterized as a bulk degradation pathway, it has become increasingly clear that it serves important roles in the selective degradation of cytoplasmic material in order to maintain homeostasis (Anding and Baehrecke, 2017; Kirkin and Rogov, 2019). Bulk autophagy is induced by nutrient deprivation and serves to replenish essential metabolites, whereas in selective autophagy substrates or cargos, such as damaged mitochondria, intracellular pathogens or aggregates, are specifically degraded to avoid the possible danger these may impose on cellular homeostasis. The importance of selective removal of damaged proteins through autophagy (aggrephagy) has become clear with the identification of mutations in autophagy receptors that cause neurodegenerative disease associated with protein aggregation (Deng et al., 2017; Rui et al., 2015). More knowledge on aggrephagy might enable the design of strategies that interfere with this specific autophagic processes and lead to novel therapies.

In autophagy, a double-membrane vesicle, the autophagosome, forms around cytoplasmic cargo. First, a crescent-shaped cisterna, the isolation membrane, is formed within omegasomes, endoplasmic reticulum (ER) subdomains enriched in phosphatoidyl-3-phosphate [PI(3)P] (Axe et al., 2008). The isolation membrane is subsequently elongated and closed to complete autophagosome formation, after which it will fuse with lysosomes to ensure degradation. Although the core components of autophagy are well known, the exact timing and spatial regulation of selective autophagy processes are not well described. Starvation-induced autophagosomes form throughout the cell and then concentrate in the perinuclear area, where they fuse with lysosomes (Jäger et al., 2004; Jahreiss et al., 2008; Kimura et al., 2008). Much less is known about the spatiotemporal dynamics of aggrephagy. For example, it is not known whether autophagosomes directly form at the site of aggregate formation, how long mature autophagosome containing an aggregate exist before fusion with lysosomes, and where this fusion occurs.

Answering these questions requires direct observation of aggrephagy. Previous work has focused on imaging of constitutive (Cason et al., 2021; Maday and Holzbaur, 2014; Maday et al., 2012) or starvation-based autophagy (Axe et al., 2008; Itakura and Mizushima, 2010; Jahreiss et al., 2008; Karanasios et al., 2016; Koyama-Honda et al., 2013; Tsuboyama et al., 2016), mitophagy (Dalle Pezze et al., 2021; Wong and Holzbaur, 2014; Zachari et al., 2019) and xenophagy (Kageyama et al., 2011). Although this has revealed the timing of recruitment of specific autophagy proteins, it has remained unclear to which extent these findings apply to aggrephagy. Moreover, in these previous studies, it remained unexplored how the successive recruitment of autophagy mediators relates to successful cargo degradation.

Recently, we introduced the particles induced by multimerization (PIM) assay as an inducible assay to study aggrephagy (Janssen et al., 2018). The PIM assay allows for the inducible formation of proteinaceous clusters inside living cells by rapalog2-induced multimerization of PIM protein, which is composed of several homodimerization domains. We have previously shown that these clusters behave as aggregates inside the cell and are targeted to the lysosome via the selective autophagy pathway (Janssen et al., 2018). This assay uniquely allows the direct observation of autophagic aggregate clearance as well as its dynamics and spatio-temporal regulation. We previously employed the dual EGFP-mCherry tag to visualize transfer of aggregates to the lysosome by the selective loss of EGFP fluorescence from the PIM clusters. However, this approach requires two optimal channels for live-cell imaging and therefore precludes straightforward labelling and high-contrast imaging of other proteins in conjunction with autophagic turnover of aggregates. Preferably, the GFP channel would be left available to facilitate the imaging of such proteins at low expression levels. Here, we describe the development of the mKeima-PIM assay, which uses a single-color pH-sensitive fluorophore to enable live-cell imaging of aggrephagy in combination with key markers of various steps in the autophagy pathway, such as the PI(3)P-binding protein DFCP1 (also known as ZFYVE1), the autophagosomal SNARE protein STX17 and the late endosomal (LE) and lysosomal marker RAB7 (also known as RAB7A). This enabled us to unravel the spatiotemporal dynamics of the autophagic clearance of aggregates.

## RESULTS

### mKeima-PIM aggregates are cleared via the autophagy pathway

**Fig. 1.**
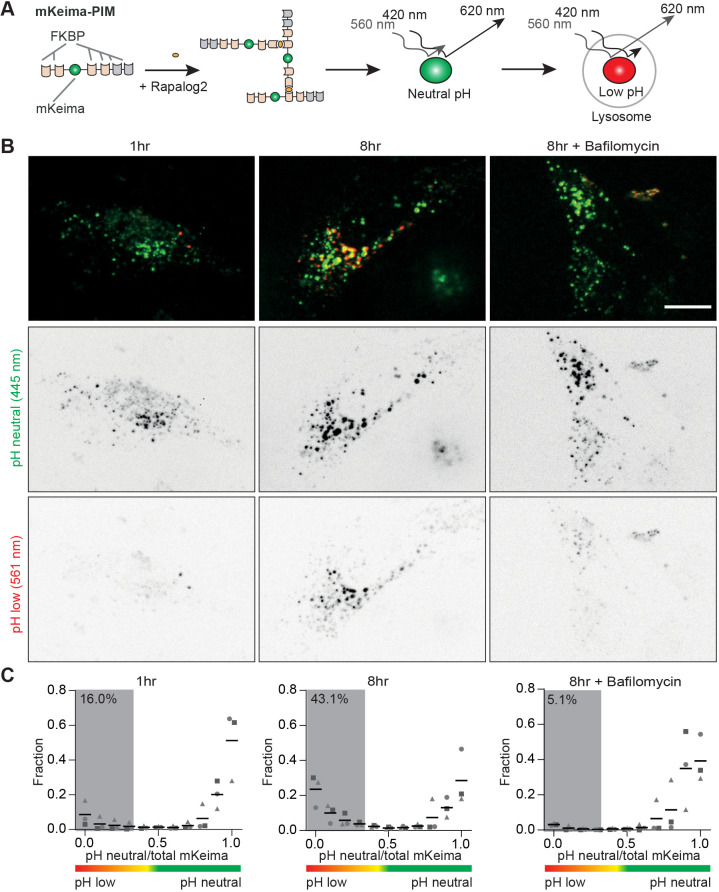
**The mKeima-PIM assay enables following aggrephagy using a single emission channel.** (A) Assay: mKeima-PIM aggregates are formed by multimerization upon rapalog2 addition. Peak emission of mKeima fluorophore is at 620 nm, but the excitation spectrum depends on pH. Aggregates are pseudo-coloured throughout this paper: green for excitation at ∼420 nm and red for excitation at ∼560 nm. (B) HeLa cells expressing mKeima-PIM at different time points after cluster formation by addition of rapalog2 (top row). Inverted contrast greyscale panels show mKeima emission at neutral pH using 445 nm excitation (middle row) and emission of mKeima at low pH with 561 nm excitation (bottom row). Cells in the third column were treated with 200 nM Bafilomycin A1. (C) Distribution of the ratio of mKeima fluorescence intensity, defined as *I*_mKeima-neutral pH_/*I*_total mKeima_ at 1 h (left) and 8 h (middle) after cluster formation, and 8 h after formation in the presence of Bafilomycin (right). For control datasets at 1 h (*n*=1258, *n*=1174 and *n*=1639 clusters) or 8 h (*n*=1549, *n*=799 and *n*=1071 clusters) and the Bafilomycin dataset (*n*=1423, *n*=533 and *n*=907 clusters), three independent experiments were analysed (indicates by circles, triangles and squares). The mean from three independent experiments is indicated. The percentage of clusters in low pH environments is indicated as the average fraction of clusters with a ratio <0.35 of three independent experiments. Scale bar: 10 µm.

To combine direct observation of aggrephagy with imaging of specific players, we decided to exchange the dual tag in the PIM construct for the mKeima fluorophore (Fig. 1A). mKeima is a pH-sensitive fluorophore with unique spectral properties that make it very useful for the autophagy field (An and Harper, 2018; Katayama et al., 2011; Lazarou et al., 2015). Specifically, mKeima emission peaks at 620 nm and, importantly, has a bimodal excitation spectrum with peaks at 440 and 568 nm. Although the emission profile remains unchanged, the efficiency of excitation is pH sensitive and shifts from 440 nm at neutral pH (cytoplasmic) to 568 nm for acidic pH (lysosome). Moreover, mKeima is, like mCherry, resistant to lysosomal proteases, and imaging can be combined with green-emitting fluorophores (Katayama et al., 2011).

First, we expressed mKeima-PIM in HeLa cells to test whether the replacement of the dual EGFP-mCherry tag with mKeima still enabled the formation and clearance of aggregates. Indeed, we observed clear aggregate formation 1 h after rapalog2 addition (Fig. 1B). When we subsequently performed dual excitation ratiometric imaging of mKeima, we observed that the efficiency of mKeima excitation with blue light (445 nm) was reduced in favour of excitation with yellow light (561 nm) for a subpopulation of aggregates at 8 h after aggregate formation. This shift in excitation sensitivity was largely prevented by treatment with Bafilomycin A1, which is a vacuolar-type H^+^-ATPase inhibitor (Yoshimori et al., 1991) and blocks autophagosome-lysosome fusion and lysosomal degradation (Yamamoto et al., 1998). Inhibition of VPS34, a phosphoinositide 3-kinase important for autophagosome formation, by SAR405 (Ronan et al., 2014) also blocked the shift of excitation sensitivity (Fig. S1). Our results therefore show that, identically to the dual EGFP-mCherry-PIM aggregates, the mKeima-PIM aggregates are cleared by autophagy.

To quantify the clearance of aggregates by autophagy, we performed automated puncta detection using the ComDet v.0.4.1 plugin for ImageJ. This plugin automatically detects puncta and subsequently quantifies fluorescence in the detected area in both channels. From these values, we get the mKeima ratio, defined as the mKeima fluorescent intensity after 440 nm excitation divided by the total mKeima emission (440 nm and 568 nm excitation). This ratio was calculated for each particle and plotted in a histogram as a fraction of particles (Fig. 1C). A ratio close to one indicates that mKeima is mostly sensitive to blue light excitation and that the aggregate resides in a neutral environment, while a low ratio indicates an acidic environment, i.e. the lysosome. When comparing samples treated with rapalog2 after 1 h or 8 h, we indeed observed an increase in the number of particles with low ratios 8 h after aggregate formation (16.0% versus 43.1%), which was largely prevented by treatment with Bafilomycin A1 (5.1%) and SAR405 (8.4% versus 55.3% in control cells) (Fig. S1).

**Fig. 2.**
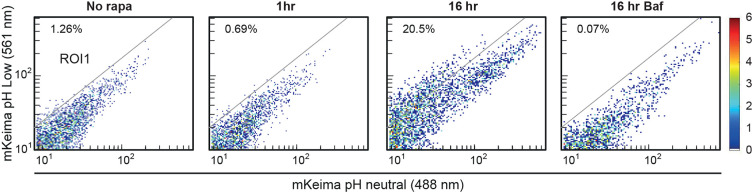
**Fluorescence-activated cell sorting (FACS)-based analysis of aggrephagy progression using mKeima-PIM.** mKeima-PIM clearance followed by flow cytometry. HeLa cells expressing mKeima-PIM were treated with rapalog2 and analysed by FACS for mKeima-PIM at low and neutral pH. Cells in the rightmost panel were treated with Bafilomycin A1. Percentage of cells in region of interest (ROI)1 is indicated.

To examine whether the mKeima-PIM assay would also be compatible with high-throughput approaches, we next measured mKeima intensity with both excitation channels in a population of cells by fluorescence-activated cell sorting (FACS) (Fig. 2). Here, 20.5% of cells displayed a spectral shift at 8 h after rapalog2 addition, compared to 0.69% at 1 h after rapalog2 addition, and this shift was completely abolished upon treatment with Bafilomycin A1. Thus, mKeima-PIM enables robust and high-throughput detection of autophagic flux following aggregate induction.

### Visualization of autophagosome formation at PIM aggregates

**Fig. 3.**
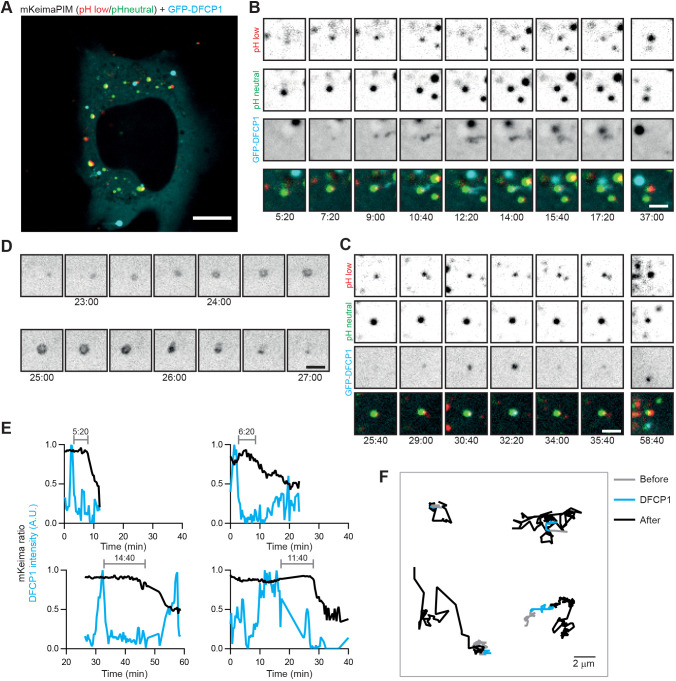
**Local omegasome formation at aggregates precedes acidification by ∼7 min.** (A) HeLa cell stably expressing GFP-DFCP1 (cyan) and transiently expressing mKeima-PIM (red and green for neutral and low pH, respectively). (B,C) Zoom of an aggregate acquiring DFCP1 signal before acidification. (D) Time-lapse imaging of ring structure formation by DFCP1. (E) Time-lapse analysis of DFCP1 intensity and mKeima ratio of individual aggregates, showing that DFCP1 is acquired before acidification. The time between presence of DFCP1 and start of mKeima-PIM acidification is indicated. (F) Analysis of mobility of aggregates before (grey), during (cyan) and after (black) DFCP1 signal was present around the aggregate. Scale bars: 10 µm (A) and 2 µm (B–D). A.U., arbitrary units.

Next, we aimed to directly study autophagosome initiation with respect to cargo in aggrephagy, and we therefore generated a stable GFP-DFCP1 cell line (Fig. 3A). DFCP1 is a PI(3)P-binding protein that localizes to omegasomes at the onset of autophagosome formation (Axe et al., 2008). During live-cell imaging at least 4 h after PIM formation, we indeed frequently observed a DFCP1-positive signal appearing at the site of an aggregate (Fig. 3B,C). Simultaneous ratiometric imaging of mKeima revealed that, in all cases, DFCP1 emerged and disappeared at the PIM site before acidification. DFCP1 disappeared from the aggregate 5–15 min before the onset of acidification (Fig. 3E). In several cases, DFCP1 was forming the ring-like structures observed previously for starvation-induced autophagy (Fig. 3D), but we typically observed smaller puncta (Fig. 3B,C). These small puncta could potentially still represent ring-like structures that could not be resolved by diffraction-limited microscopy. Interestingly, most aggregates were largely immobile before and during the presence of the DFCP1 signal, but became increasingly mobile after loss of DFCP1 from the aggregate, which coincides with the release of newly formed autophagomes from the omegasome (Fig. 3F). These observations suggest that aggregates might be tethered to a potential autophagosome formation site before and during autophagosome formation.

**Fig. 4.**
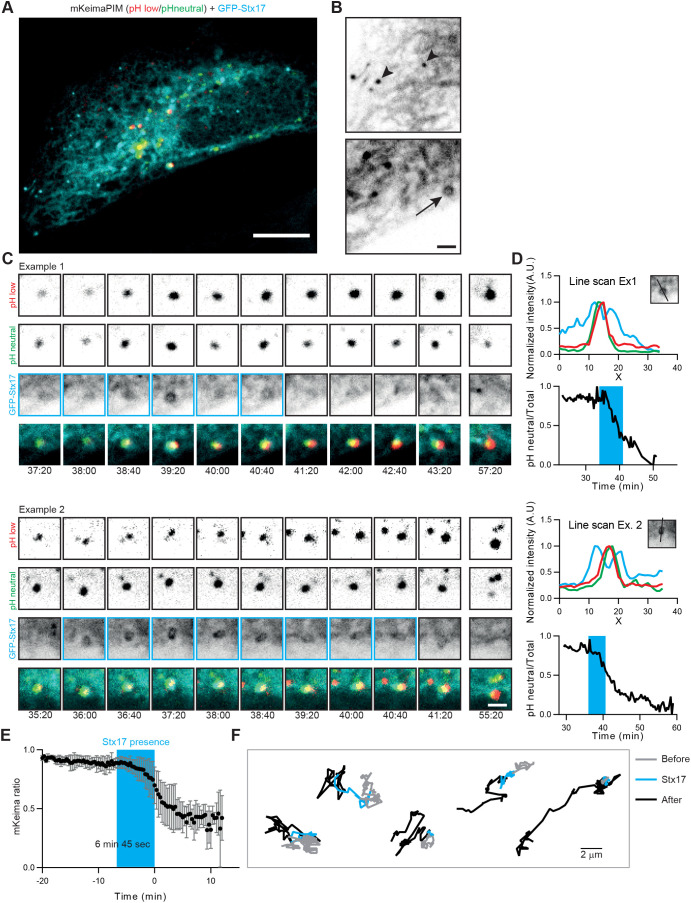
**Local autophagosome formation at aggregates is directly followed by acidification.** (A) HeLa cell stably expressing GFP-STX17 and transiently expressing mKeima-PIM. (B) Zoom of the cell in A, showing GFP-STX17 localization to endoplasmic reticulum (ER) and mitochondria, and the presence of small vesicles (arrowheads) and rings (arrow) positive for STX17. (C) Two examples with selected frames from a time-lapse movie showing STX17 recruitment to the aggregate and subsequent loss of STX17 concomitant with acidification. (D) Analysis of the time-lapse examples in C, showing intensity profiles indicating presence of the aggregate inside a STX17-positive autophagosome (top) and the mKeima ratio over time (bottom). The blue boxes indicate when a STX17-positive signal at the aggregate was observed. (E) Average mKeima ratio over time and indication of average STX17 signal presence. Data represent mean±s.d. from 12 aggregates from two independent experiments. Events were aligned according to the first frame after STX17 disappearance. (F) Analysis of mobility of aggregates before (grey), during (cyan) and after (black) STX17 signal was present around the aggregate. Scale bars: 10 µm (A) and 2 µm (B,C).

To visualize completion of autophagosome formation, we generated a stable GFP-STX17 cell line. STX17 is the autophagosomal SNARE involved in fusion of the autophagosome with LE or lysosome (Itakura et al., 2012), and is recruited at the end of autophagosome formation, probably by mATG8s (Kumar et al., 2018). STX17 is not recruited to the isolation membrane and is therefore a useful marker to identify fully formed autophagosomes (Itakura et al., 2012). In line with previous observations (Itakura et al., 2012), we observed STX17 localization to the ER and mitochondria under normal nutritional conditions (Fig. 4A,B; Fig. S2A–D). In addition, we observed punctate (arrowheads) and ring-shaped (arrow) structures that suggested the presence of autophagosomes >4 h after aggregate induction (Fig. 4B).

### Autophagosomes remain mostly immobile until acidification

During time-lapse imaging of preformed aggregates (4–8 h after rapalog2 addition), we clearly observed the formation of STX17-positive rings around aggregates just before acidification of the cargo (Fig. 4C,D; Movie 1). Following the onset of acidification, STX17 was released from the aggregate-containing autophagosomes (Fig. 4D). On average, STX17 was associated with aggregates for ∼7 min. At the time of STX17 disappearance, the acidification ratio had proceeded to ∼30% of the total change (Fig. 4E). This suggests that, once fully formed, autophagosomes quickly fuse with LEs or lysosomes. Moreover, by monitoring the displacements before, during and after STX17 association, we found that aggregates remain largely immobile before and during the presence of STX17 (Fig. 4F). Only after STX17 disappearance, marking the fusion with LE or lysosome, motility is increased. Although the conventional view holds that autophagosomes are transported to lysosomes in order to form autolysosomes, these results demonstrate that, in aggrephagy, the reverse scenario is more likely. The nascent autophagosomes remain immobile and potentially tethered until acidification, when amphisome or autolysosome formation has occurred through fusion with LEs or lysosomes, respectively.

### STX17 recruitment does not always lead to successful acidification

**Fig. 5.**
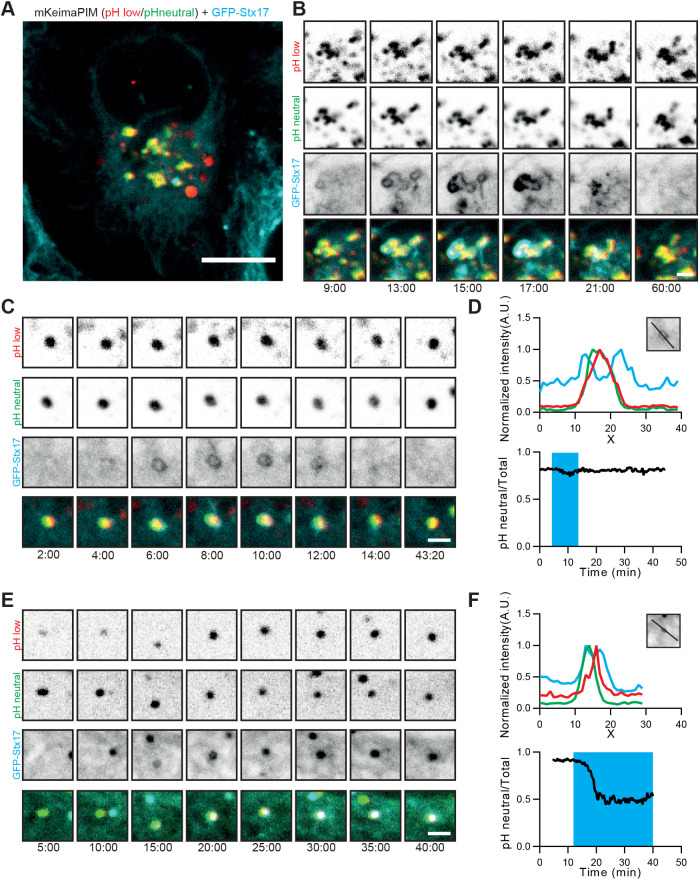
**STX17 recruitment does not always result in successful clearance.** (A) A HeLa cell stably expressing GFP-STX17 and transiently expressing mKeima-PIM. (B) Selected frames from a time-lapse movie showing STX17 recruitment and dissociation from a larger aggregate not followed by acidification. (C) Example showing frames from a time-lapse movie in which STX17 recruitment did not lead to subsequent acidification. (D) Analysis of the time-lapse examples in C, showing the presence of the aggregate inside the STX17-positive autophagosome and the mKeima ratio over time. The blue box indicates the time when a STX17-positive signal at the aggregate was observed. (E) Frames from a time-lapse movie showing prolonged recruitment of STX17 leading to partial but not full acidification. (F) Analysis of the example shown in E with an intensity profile and the mKeima ratio over time. The blue box indicates the time when a STX17-positive signal at the aggregate was observed. Scale bars: 10 µm (A) and 2 µm (B,C,E).

When expressed at higher levels, bigger aggregates can form with more amorphous structures than the largely spherical aggregates we described so far. To see whether autophagosomes could also form around these bigger structures, we imaged STX17 together with these larger mKeima-PIM aggregates (Fig. 5A). Interestingly, although we did observe the association of STX17 with these big aggregates (Fig. 5B), there was no clear indication that STX17 release coincided with autophagosome maturation and subsequent degradation. The dissociation of STX17 from the aggregate is believed to indicate successful breakdown of the inner autophagosomal membrane (IAM) (Tsuboyama et al., 2016) and thereby the onset of degradation and acidification. Nonetheless, we did not observe any mKeima shift in these aggregates, which raises the question how and why STX17 leaves these autophagosomal structures. One possibility would be that STX17 was erroneously recruited before autophagosome closure, which has been observed previously (Tsuboyama et al., 2016). This is supported by our observation that, also for smaller aggregates, not every STX17 recruitment event led to degradation. In several cases, we observed either the loss of STX17 without concomitant acidification (Fig. 5C,D) or the stable presence of STX17 signal on aggregates (Fig. 5E,F). In the latter case, STX17 remained localized to the aggregate even though acidification of the autophagosome had already started, based on the decrease in the mKeima ratio. Based on the earlier finding that IAM breakdown, rather than acidification, causes STX17 dissociation (Tsuboyama et al., 2016), this suggests that IAM breakdown is delayed or blocked.

### Full autophagosome acidification occurs through multiple contact and fusion events

**Fig. 6.**
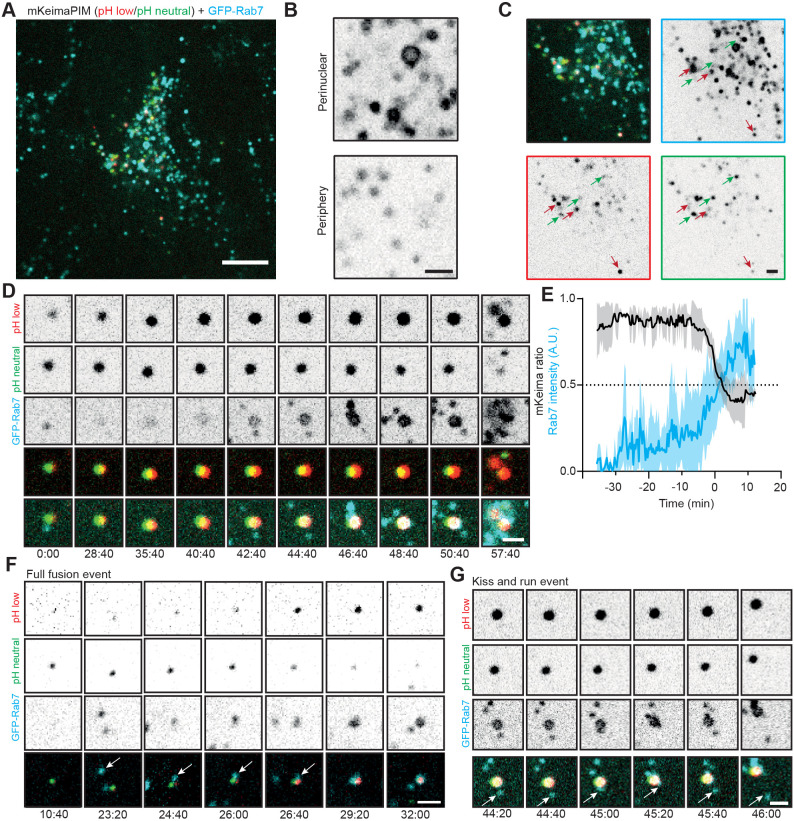
**RAB7 is gradually recruited to aggregates through multiple fusion and contact events.** (A) Endogenous GFP-RAB7 KI HeLa cell line transiently showing mKeima-PIM aggregates. (B) Endogenous GFP-RAB7 signal showing larger RAB7-positive vesicles at the perinuclear area and smaller vesicles in the periphery. (C) Zoom of the cell in A, showing aggregates at neutral pH (green arrows) and low pH (red arrows) and correlation with RAB7. (D) Selected frames from a time-lapse movie showing the gradual recruitment of RAB7 and concomitant decrease of mKeima ratio. (E) Analysis of RAB7 intensity (cyan) and mKeima ratio (black) of ten typical acidification events, including the one shown in D. Data shown are mean (solid line) ±s.d. (shaded area) of three to ten events from seven independent experiments. (F,G) Time-lapse imaging of GFP-RAB7 KI HeLa cells showing putative kiss-and-run events with RAB7-positive vesicles (G) and full fusion event of RAB7-positive vesicle with the autolysosome (F). Scale bars: 10 µm (A) and 2 µm (B–D,F,G).

The final steps of autophagy involve multiple fusion and kiss-and-run events with endosomes and lysosomes to enable acidification and final degradation of the autophagosomal content (Dunn, 1990; Jahreiss et al., 2008; Zhao and Zhang, 2019). RAB7 is a member of the Rab family of small GTPases and localizes to LE and lysosome (Guerra and Bucci, 2016; Jimenez-Orgaz et al., 2018). To directly examine the interplay between aggregate-containing autophagosomes and both LEs and lysosomes, we tagged endogenous RAB7 with GFP using CRISPR-Cas9-mediated genome editing (Fig. S3A,B). The generated GFP-RAB7 cell line clearly shows vesicular structures ranging in size with bigger vesicles close to the nucleus and smaller vesicles at the periphery (Fig. 6A,B). Aggregates with low mKeima ratios (low pH, red arrows; Fig. 6C) were typically associated with higher RAB7 levels than aggregates with high mKeima ratios (high pH, green arrows; Fig. 6C). When we monitored an aggregate over time, we often observed multiple contacts with RAB7-positive vesicles and a gradual increase in RAB7 signal, together with a decrease in mKeima ratio (Fig. 6D,E). RAB7-positive vesicles that contacted the forming autolysosome would sometimes fuse (Fig. 6F; Movie 2), but most often left after several seconds, reflecting potential kiss-and-run events to transfer membrane-bound proteins and/or proteolytic enzymes (Fig. 6G). In perinuclear regions crowded with RAB7-positive vesicles, forming autolysosomes showed multiple rounds of transient contact with RAB7, eventually resulting in content acidification (Fig. S4A,B, Movie 3). These observations demonstrate that multiple contact events and fusions are often needed for the full acidification and autophagosomal degradation of aggregates.

## DISCUSSION

**Fig. 7.**
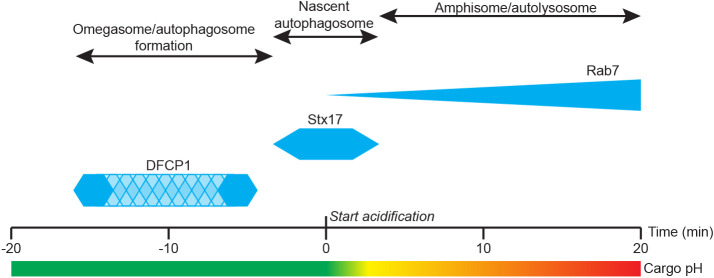
**Timing of autophagy factor recruitment with respect to aggregate acidification.** First, DFCP1 is recruited to aggregates (omegasome formation). DFCP1 puncta are present only for a short time, but the timing of DFCP1 puncta presence relative to acidification is variable (patterned area). Just before acidification of the cargo, STX17 is recruited for ∼7 min, and STX17 leaves the autophagosome after the start of cargo acidification. RAB7 is gradually recruited to the autophagolysosome while cargo acidification is also taking place.

Here, we have introduced the mKeima-PIM assay as an inducible probe for the autophagic clearance of aggregates. Compared to the dual-PIM assay that we previously introduced, the mKeima-PIM assay offers the important advantage that it reports on acidification using only the red emission channel, enabling the combined imaging with GFP-labelled proteins. This facilitates the use of cell lines with stably expressing GFP-labelled proteins and enabled us to directly visualize aggregate clearance in conjunction with different key autophagy factors. Taken together, our results demonstrate that, during aggrephagy, the isolation membrane starts forming from the omegasome at the aggregate site ∼5–15 min before start of cargo acidification (Fig. 7). The lifetime of the omegasome itself is only a few minutes. Completion of autophagosome formation, as identified by STX17 recruitment, is in most cases directly followed by autophagosome acidification. The mature autophagosome has an average lifetime of ∼7 min before acidification and STX17 dissociation. Finally, RAB7 gradually accumulates on the amphisome or autolysosome by multiple kiss-and-run and full fusion events, resulting in a drop in pH and final cargo degradation.

Previous work has used live-cell imaging to analyse the temporal sequence in which different autophagy-related proteins are recruited to autophagic sites following starvation (Koyama-Honda et al., 2013). This revealed that p62 (also known as SQSTM1), a receptor and substrate for selective autophagy, accumulated at autophagic sites after appearance of DFCP1, suggesting that substrates for autophagy are transported to preformed omegasomes. In contrast, our results show that the omegasome (which is DFCP1 positive) is formed at the cargo site. This suggests that autophagosome formation is, at least partially, instructed by the aggregates. Indeed, cargo-instructed autophagy was recently proposed for selective mitophagy, in which accumulation of the autophagy receptor NDP52 (also known as CALCOCO2) on mitochondria leads to localization and activation of the ULK1 complex to induce autophagosome formation (Vargas et al., 2019). Recruitment of upstream autophagy machinery by autophagy receptors was also proposed for xenophagy (Ravenhill et al., 2019) and p62 condensates (Turco et al., 2019) and resembles the situation described for the Cvt pathway in yeast, in which, unlike non-selective autophagy, the cargo is required for proper autophagosome formation (Shintani and Klionsky, 2004). Such a cargo-instructed process could facilitate the selective clearance of specific items and prevent engulfment of other cytoplasmic factors (Rogov et al., 2014; Sawa-Makarska et al., 2014; Zaffagnini and Martens, 2016).

Using our live-cell approach, we found that aggregate-containing autophagosomes are relatively short-lived structures and that fully formed autophagosomes only persist for ∼7 min before STX17 dissociation and complete acidification of the autolysosome. This is consistent with the lifetime of STX17-positive autophagosomes in starvation conditions (Tsuboyama et al., 2016). In addition, we could demonstrate that maturation into autolysosomes or amphisomes and the corresponding acidification typically takes place at the site of autophagosome formation through multiple fusion or kiss-and-run events. Thus, in contrast to what has been shown for starvation-induced autophagy (Jahreiss et al., 2008), aggregate-triggered autophagosomes are largely immobile and depend on motile lysosomes and late endosomes for acidification near their site of formation.

In comparison to earlier work that performed live-cell imaging of autophagy, an important advantage of mKeima-PIM is that it enables simultaneous probing of cargo fate and different components of the autophagy machinery. This revealed, for example, that often the inner part of the autophagosome already starts to acidify in the presence of STX17, presumably before IAM breakdown. In addition, it revealed that recruitment and release of STX17 is not always a predictor of successful acidification.

In addition to providing new imaging opportunities, the use of mKeima was also beneficial for FACS. Using dual EGFP-mCherry-PIM, we previously reported that a fraction of PIMs displayed a spectral change in the presence of Bafilomycin A1, most likely as a result of GFP self-quenching (Janssen et al., 2018). In contrast, using mKeima-PIM, we observed no spectral changes in this condition, and we therefore recommend this approach for high-throughput analysis of autophagy using FACS. Nonetheless, for image-based analysis of autophagy progression we recommend using the dual EGFP-mCherry-PIM in fixed samples. Robust ratiometric imaging can be hampered by aggregate displacement within the time required to take two sequential images. As such, analysing fixed samples would enable more reliable analysis. Because the pH gradient across the lysosomal membrane is lost upon fixation, mKeima cannot be used in fixed samples. In the dual EGFP-mCherry-PIM assay, GFP is degraded and therefore the colour switch observed on lysosomal transfer is preserved upon fixation. The two PIM assays thus have benefits for different types of experiments, which together will likely lead to a deeper understanding of aggrephagy.

## MATERIALS AND METHODS

### Plasmids

The mKeima-PIM construct was an adapted version of our PIM construct published before (Janssen et al., 2018). We exchanged the mCherry and EGFP for mKeima, resulting in a construct consisting of four FKBP homodimerization domains with sequence variation, mKeima and two FKBP heterodimerization domains in the mammalian expression vector pβactin. The first two FKBP homodimerization domains contain three mutations (V24E, Y80C and A94T) that were found to aid multimerization. mKeima was amplified by PCR using mKeima-RED-N1 (Addgene #54597) as a template. mKeima-RED-N1 was a gift from Michael Davidson (Florida State University, Tallahassee, Florida).

pDONOR-GFP-RAB7 encodes a tagging module, consisting of a 6xGGGGS linker and an N-terminal EGFP tag, flanked by 1000 bp homology arms that are homologous to the genomic region immediately surrounding the RAB7 start codon, and was generated by PCR and Gibson assembly strategies. Primers used to amplify the 5′ homology arm were 5′-GGAGATCGGTACTTCGCGAATGCGTCGAGATGCGGCCGCTACCCTGGCAAATGAGAGGC-3′ and 5′-TCCGTTCCAGTGTGGTTGCCAGCATGGTGGCGCGCCCCTTCAAACTAAAGGGGGAAAAG-3′. Primers used to amplify the 3′ homology arm were 5′-GGAGGGGGTTCTGGTGGTGGTAGCTACGTAACCTCTAGGAAGAAAGTGTTGCTG-3′ and 5′-TGCACTCGTCGGTCCCGGCATCCGATACGCGTGCGGCCGCCTGGGCAAATGCTAGCGAAC-3′.

For generation of stable cell lines, EGFP and DFCP1/STX17 were cloned into the pLVX-IRES-Hygro vector (a gift from Harold MacGillavry, Utrecht University, Utrecht, The Netherlands) using the EcoRI and SpeI restriction sites. DFCP1 and STX17 were amplified by PCR using mCherry-DFCP1 (Kim et al., 2015) and FLAG-STX17 (Itakura et al., 2012) as templates. FLAG-STX17 was Addgene #45911, deposited by Noboru Mizushima, and mCherry-DFCP1 was Addgene #86746, deposited by Do-Hyung Kim.

### Cell culture and transfection

HeLa cells and HEK293T cells were cultured in Dulbecco's modified Eagle medium containing 10% foetal calf serum and penicillin/streptomycin. Cells were maintained at 37°C and 5% CO_2_ and were frequently tested for mycoplasma contamination using MycoAlert Mycoplasma Detection Kit (Lonza). HeLa cells were purchased from American Type Culture Collection (ATCC) and used as wild-type cells or modified for stable expression of STX17, DFCP1 or RAB7. HEK293T cells were a gift from Anna Akhmanova (Utrecht University, Utrecht, The Netherlands). Cells were not formally authenticated.

Cells were plated on 25-mm diameter coverslips and transfected using Fugene6 transfection reagent (Roche), according to the manufacturer's protocol. Experiments were started 1 day after transfection.

### Generation of stable cell lines

Cells stably expressing GFP-DFCP1 and GFP-STX17 were generated using a lentiviral expression system. Lentivirus packaging was performed using MaxPEI-based co-transfection of HEK293T cells with psPAX2 (Addgene #12260), pMD2.G (Addgene, #12259) and the lentiviral vector pLVX-IRES-Hygro-EGFP-DFCP1 or pLVX-IRES-Hygro-EGFP-STX17. Supernatant of packaging cells was harvested up to 72 h of transfection, filtered through a 0.45-µm filter and incubated with a polyethylene glycol (PEG)-6000-based precipitation solution overnight at 4°C. After precipitation, virus was concentrated up to 100× by centrifugation and dissolution in 1× phosphate buffered saline (PBS). HeLa cells were incubated for 4 h in complete medium supplemented with 8 µg/ml polybrene before infection. To establish stable HeLa cell lines carrying EGFP-tagged DFCP1 or STX17, medium was replaced 24–48 h after infection and 100 µg/ml Hygromycin (Invitrogen, ant-hg-5) was added.

Endogenous tagging of RAB7 was performed by CRISPR-Cas9-mediated genome editing (Ran et al., 2013). The HeLa GFP-RAB7 line was generated by transfecting HeLa cells (ATCC) with the donor plasmid pDONOR-GFP-RAB7 and pSpCas9(BB)-2A-Puro (PX459) V2.0 (Addgene, #62988) bearing the appropriate targeting sequence (5′-TAGTTTGAAGGATGACCTCT-3′; pX459v2-RAB7a sg3). Then, 24 h after transfection, Cas9-positive cells were selected by treatment with 1 µg/ml puromycin for 64 h. Subsequently, cells were grown to confluency and seeded as single cells. EGFP-Rab7-KI was validated by PCR amplification (using FW, 5′-TACCCTGGCAAATGAGAGGC-3′ and RV, 5′-CTGGGCAAATGCTAGCGAAC-3′) and sequencing (5′-GGCTTAGCTCTAAGCCAATC-3′). Single clones were selected to have correct labelling of RAB7 by live-cell microscopy and colocalization analysis with endo-lysosomal markers by immunofluorescence microscopy and were confirmed by sequence analysis.

### Fluorescence microscopy

Live-cell imaging was performed on a Spinning Disc Nikon Eclipse Ti Microscope with Perfect Focus System controlled by MetaMorph 7.7 software (Molecular Devices). An incubation chamber (Tokai Hit, INUBG2E-ZILCS) was used mounted on a motorized stage (ASI, MS-2000-XYZ). Coverslips were mounted in metal imaging rings immersed in medium. During imaging, cells were maintained at 37°C and 5% CO_2_. Cells were imaged every 20 s for 1 h using a 60× oil immersion objective (Plan Apo VC, 1.4 NA, Nikon) and an Evolve 512 EMCCD camera (Photometrics). Vortran Stradus 445 nm, Cobolt Calypso 491 nm and Cobolt Jive 561 nm lasers were used for excitation. Aggregates were formed by addition of 500 nM rapalog2 (Takara, B/B homodimerizer #635059) for 1 h, and cells were imaged 2–8 h after aggregate formation. Analysis of stable lines only extended to GFP-positive cells.

For imaging of cells at specific time points, aggregates were induced and rapalog2 was washed out after 1 h, after which cells were imaged at 1 h or 8 h time points by taking 3 *z*-planes, 200 nm apart. Cells were incubated with 200 nM Bafilomycin A1 or 10 μM SAR405 (Selleckchem, S7682) for 30 min before rapalog2 addition. All drugs were reapplied after rapalog2 washout.

### Immunofluorescence cell staining, imaging and antibodies

For validation of GFP-STX17 localization in the stable HeLa GFP-STX17 cell line, cells were fixed with 4% paraformaldehyde for 10 min at room temperature (RT). Cells were washed in PBS, permeabilized using 0.2% Triton X-100, and blocked using 3% bovine serum albumin in PBS. Cells were incubated for 1 h at RT with primary antibodies. Cells were subsequently washed in PBS before incubation with secondary antibodies for 1 h at RT. Cells were washed in PBS and mounted using Prolong Diamond (Thermo Fisher Scientific). Images were taken on a Nikon Eclipse Ni-U microscope with a 100× oil immersion objective (Plan Apo Lambda, 1.44 NA, Nikon). Illumination was done with CoolLED pE-300 using ET-EGFP (49002, Chroma) and ET-mCherry (49008, Chroma) filter sets.

The following primary antibodies were used: mouse anti-TOM20 (Becton Dickinson, Cat. No. 612278, Lot No. 9204806, 1:500), rabbit anti-calnexin (Abcam, Cat. No. ab22595, Lot No. GR3359380-1, 1:500) and chicken anti-GFP (Aves Labs, Cat. No. GFP-1010, Lot No. GFP3717982, 1:250). Appropriate Alexa Fluor-conjugated secondary antibodies were used at 1:500 dilution (Life Technologies, Cat. No. A11031, Lot No. 2026148; Cat. No. A11036, Lot No. 2045347; Cat. No. A11039, Lot No. 1869581).

### Image analysis

For analysis of different time points, first an average *z*-projection was made from the stack using ImageJ (National Institutes of Health). A region of interest (ROI) was placed around the cells, and the ComDet v.0.4.1 plugin (https://github.com/ekatrukha/ComDet) was used to detect particles using an approximated particle size of 4 pixels and a signal-to-noise ratio of 8 for both mKeima channels. Data were transferred to Microsoft Excel, where the mKeima ratio per particle was calculated as the mKeima integrated intensity with 445 nm excitation divided by the total integrated intensity (at 445 nm and 561 nm excitation). Frequency distribution graphs were plotted using GraphPad Prism7 software. Graphs represent data from three independent experiments, with 10–15 cells per experiment. The percentage of cleared clusters is calculated as the average percentage of three independent experiments of clusters with an mKeima ratio <0.35 (sum from histogram frequencies with bin 0–0.3).

For analysis of individual cluster acidification, an ROI was placed around the particle, and intensity was measured in both mKeima channels. Background intensities were measured in regions of the cell in which no clusters were present. Data were transferred to Microsoft Excel, and, after background subtraction, the mKeima ratio was calculated. Tracking of aggregates was performed by manual tracking using ImageJ.

The time interval between DFCP1 presence at the aggregate and acidification was calculated by taking the difference between the time at which the normalized DFCP1 signal dropped and stayed below 0.7 of the maximum intensity and the mKeima ratio stayed below 0.8.

For the averaged timing of aggregate acidification and STX17 presence, all the traced aggregates were aligned according to the first frame without STX17 presence as scored manually.

For analysing average RAB7 intensity during aggregate acidification, individual events were aligned according to the frame at which mKeima ratio dropped halfway to the minimum value. Time points at which aggregates were out of focus or overlapped were not included in the analysis.

### FACS

FACS analysis was performed on a BD Influx Cell Sorter. Measurements were made using a 488 (pH neutral) and 561 (pH low) nm laser with 630/22 nm and 610/20 nm emission filters, respectively. For each sample, 20,000 events were collected and subsequently gated for singlets and mKeima-positive cells. Data were analysed using FlowJo v10 and plotted using MATLAB and GraphPad.

## Supplementary Material

Supplementary information

Reviewer comments
