# Supplementary material for: Direct observation of aggregate-triggered selective autophagy in human cells
Source: J Cell Sci. 2021 Oct 6;134(19):jcs258824. doi: 10.1242/jcs.258824 (PMC8520732; doi:10.1242/jcs.258824)
Supplement: Supplementary information [file joces-134-258824-s1.pdf]

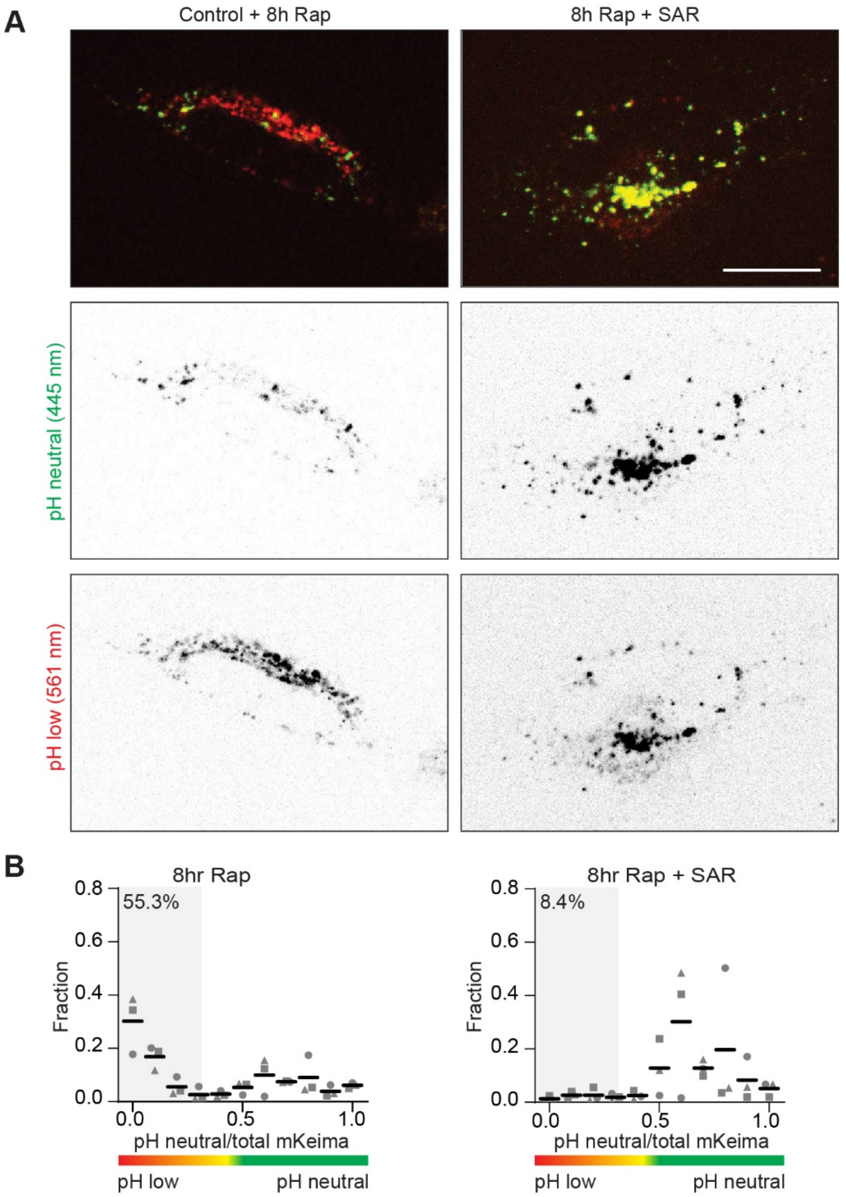

Fig. S1. Inhibition of VPS34 blocks mKeima-PIM clearance. A) HelaWT cells expressing mKeima-PIM after 8 hours of cluster formation by addition of rapalog2. Inverted contrast grayscale panels show mKeima emission at neutral pH by using 445 nm excitation (middle row, green) and emission of mKeima at low pH by 561 nm excitation (bottom row, red). Cells in the right column were treated with 10 µM SAR405 (SAR). B) Plot shows the distribution of the ratio of mKeima fluorescence intensity, defined as $I_{\text{mKeima-neutral pH}}/I_{\text{total mKeima}}$ in control cells (left) and SAR treated cells (right). Data obtained from 3 independent experiments (represented by circles, triangles and squares). Datasets contain 747, 671 or 581 clusters (8hr Rap) or 310, 282 and 252 clusters (8hr Rap + SAR). Bars represent the mean. The percentage of clusters in low pH environments is indicated as the average fraction of clusters with a ratio <0.35 of 3 independent experiments. Scale bar, 20µm.

Journal of Cell Science • Supplementary information

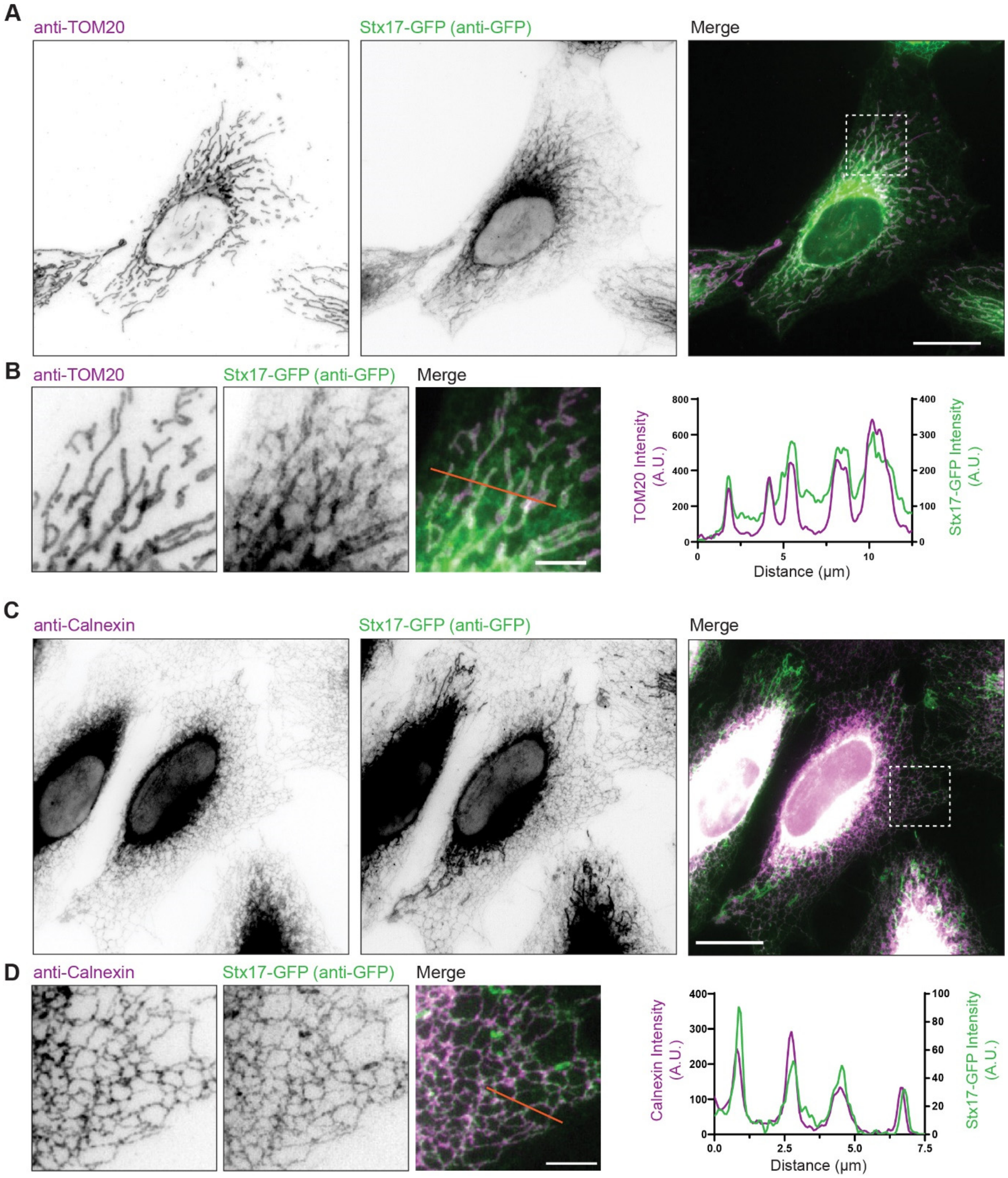

Fig. S2. Colocalization of Stx17-GFP with mitochondria and ER in Hela-Stx17-GFP stable cell line. A, C) Inverted grey scale images of immunofluorescent staining of Hela-Stx17-GFP cells stained for Stx17-GFP (green) and TOM20 (A; magenta) and Calnexin (C; magenta). B) Enhanced contrast zoom of panel depicted in (A) with line scan (orange) of TOM20 and Stx17-GFP intensity. D) Enhanced contrast zoom of panel depicted in (C) with line scan (orange) of Calnexin and Stx17-GFP intensity. Scale bars: 20µm (A, C), 5µm (B, D).

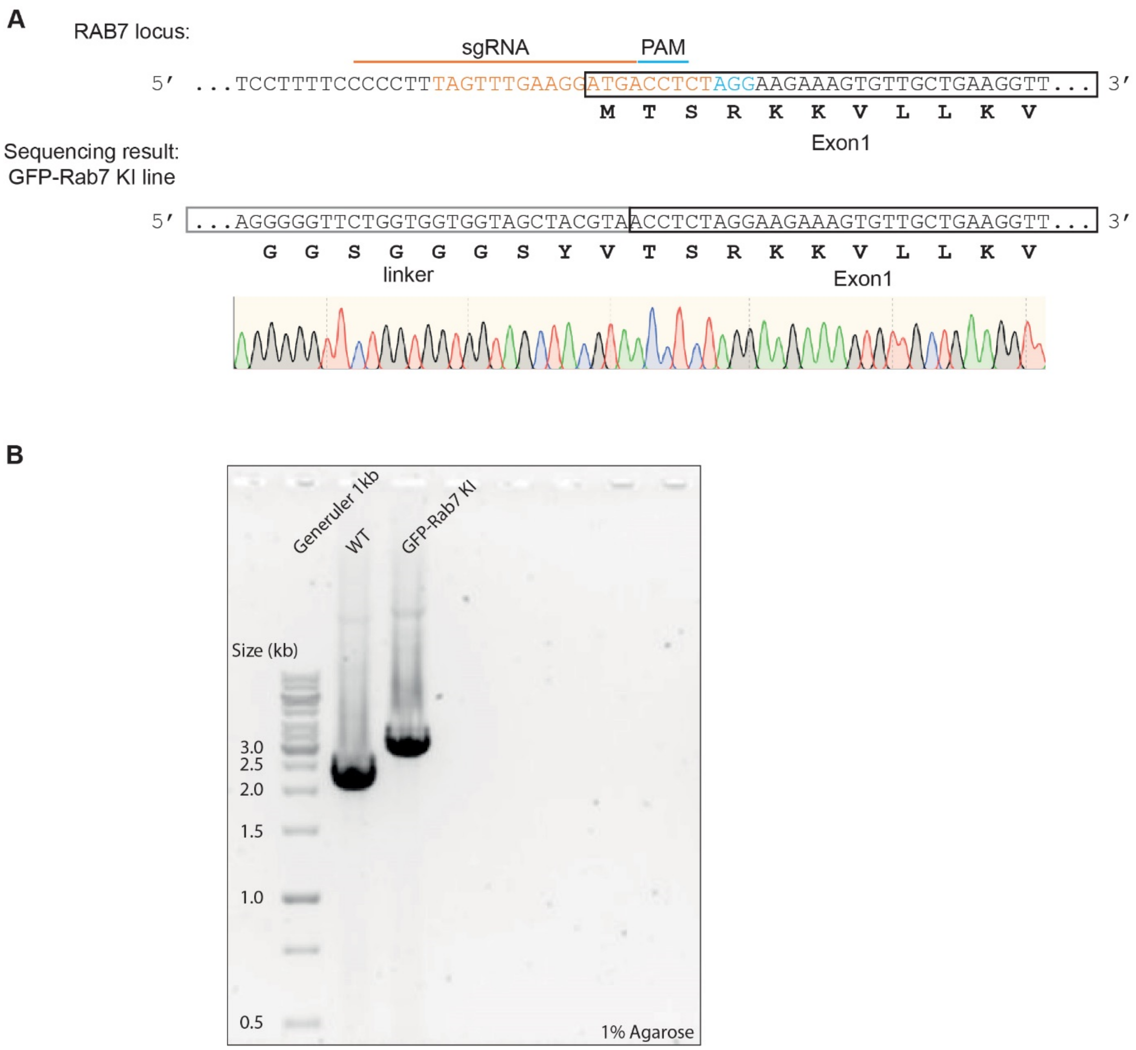

Fig. S3. GFP-Rab7 KI HeLa line validation

A) Coding strand of knock-in target site before exon 1 (black box) of the *RAB7A* locus. The sgRNA target site (orange) and PAM sequence (cyan) are indicated. Insert is EGFP followed by a linker sequence (grey box). DNA sequencing of the homozygous knock-in clone is aligned below. B) 1% agarose gel including PCR-amplification product of *RAB7A* locus of *WT* and EGFP-Rab7 KI lines.

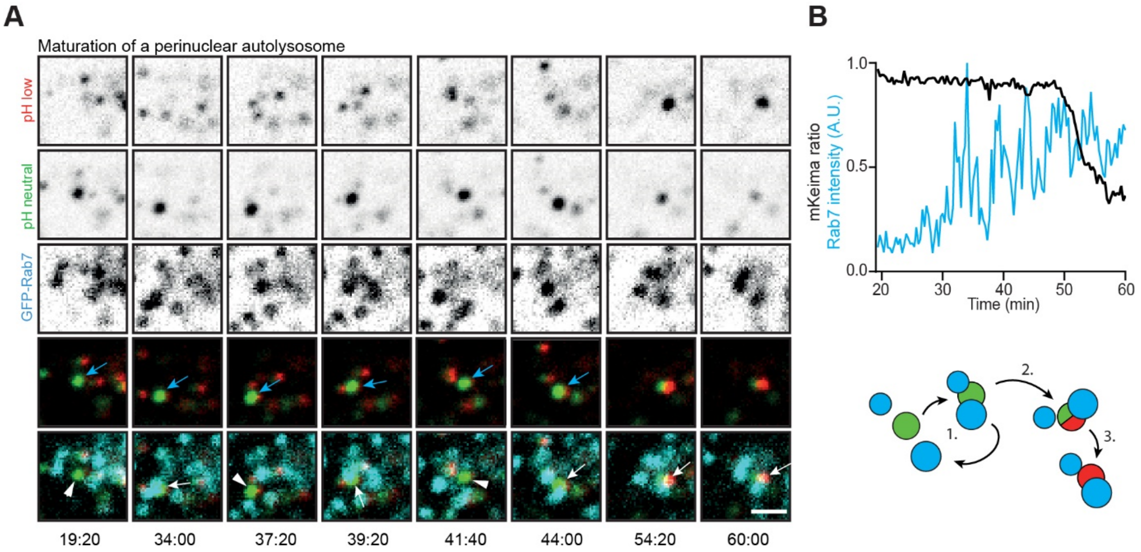

Fig. S4. Maturation of a perinuclear autolysosome. A) Monitoring a forming autolysosome in the perinuclear region (see schematic in B). Frames from a time-lapse movie depicting multiple rounds of transient associations with RAB7 positive vesicles (1.), subsequent fusion (2.) and full acidification (3.). Arrows and arrowheads indicate interactions and absence of interactions, respectively. B) Analysis of RAB7 intensity and mKeima ratio of (A). Scale bar: 2 μm

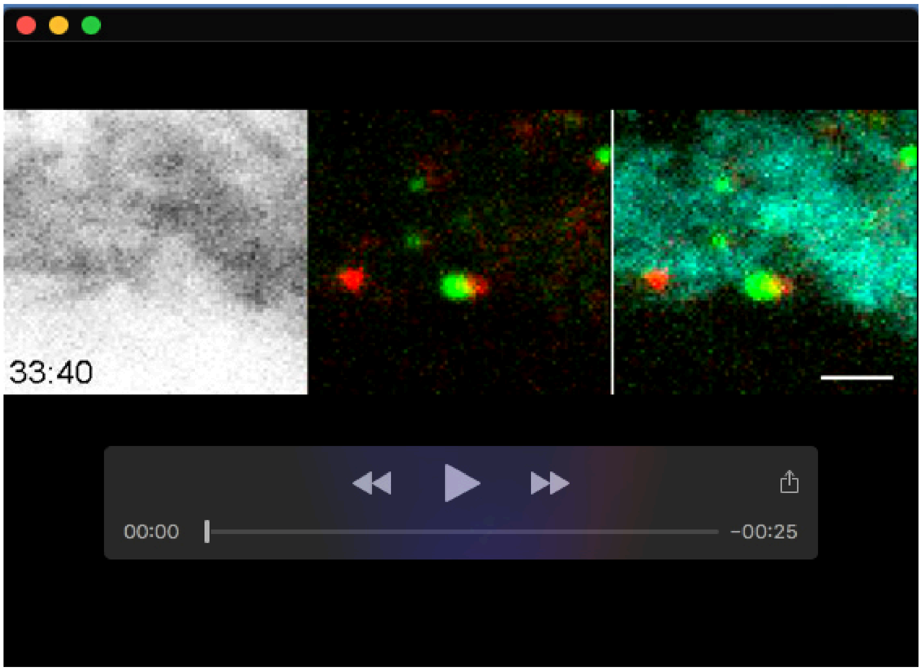

Movie 1. This video complements Fig. 4C and shows transient STX17 (left) accumulation at mKeima-PIMs (middle) with concomitant acidification marked by green to red conversion of mKeima. Time denotes minutes:seconds. Scale bar marks 2 µm.

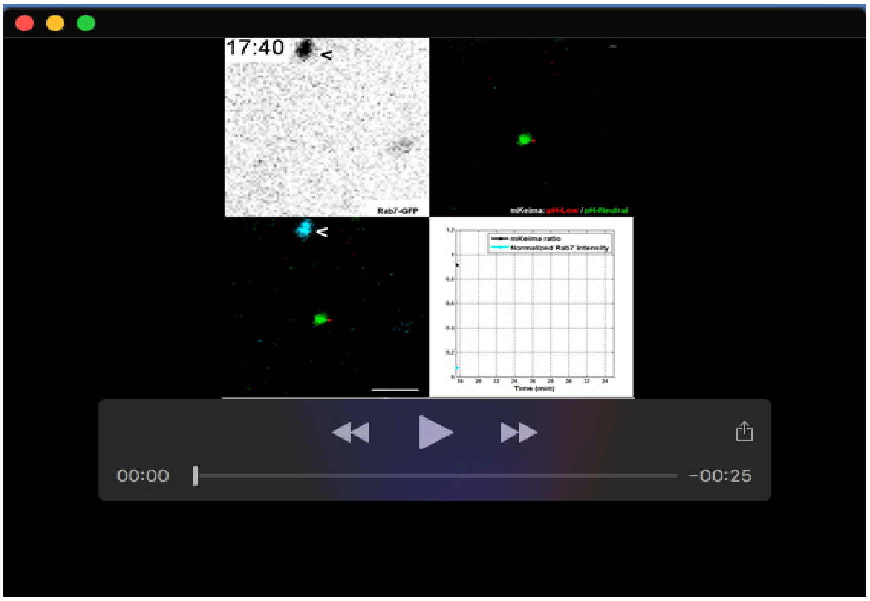

Movie 2. This video complements Fig. 6F and shows Rab7 (top left) accumulation at mKeima-PIMs (top right) with concomitant acidification marked by green to red conversion of mKeima. Time denotes minutes:seconds. Scale bar marks 2 µm.

Journal of Cell Science • Supplementary information

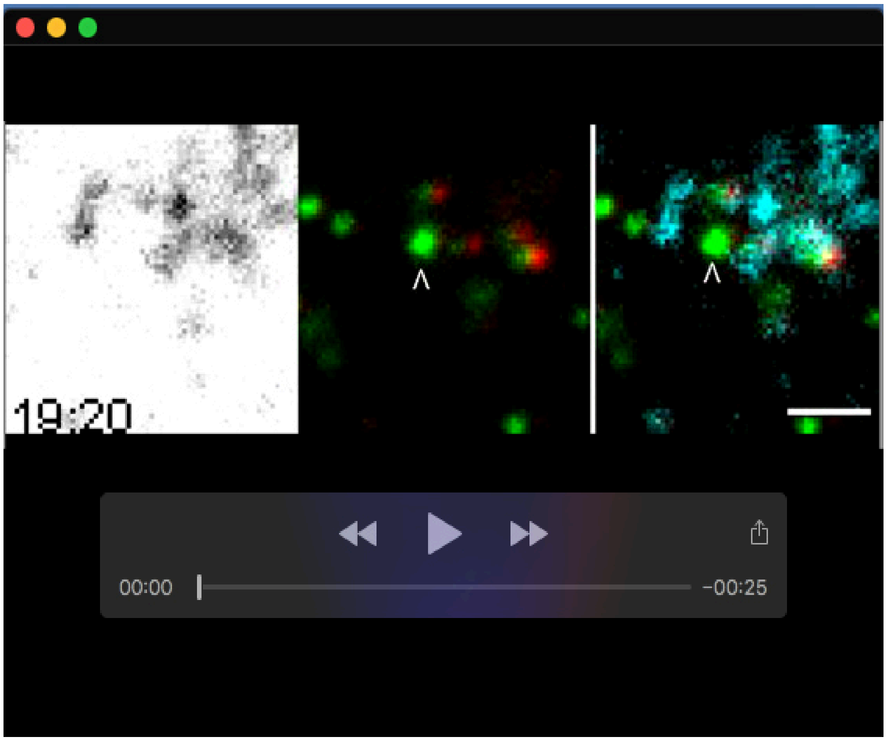

Movie 3. This video complements Fig. S4A and shows multiple rounds of transient associations of mKeima-PIM with RAB7 positive vesicles with concomitant gradual acidification. Time denotes minutes:seconds. Scale bar marks 2 µm.

Journal of Cell Science • Supplementary information
