# Supplementary material for: Direct observation of aggregate-triggered selective autophagy in human cells
Source: J Cell Sci. 2021 Oct 6;134(19):jcs258824. doi: 10.1242/jcs.258824 (PMC8520732; doi:10.1242/jcs.258824)
Supplement: Reviewer comments [file joces-134-258824_review_history.pdf]

Direct observation of aggregate-triggered selective autophagy in human cells

Anne F.J. Janssen, Giel Korsten, Wilco Nijenhuis, Eugene A. Katrukha and Lukas C. Kapitein
DOI: 10.1242/jcs.258824

Editor: Jennifer Lippincott-Schwartz
Review timeline

Original submission:	21 April 2021
Editorial decision:	3 June 2021
First revision received:	29 July 2021
Accepted:	23 August 2021

Original submission

First decision letter

MS ID#: JOCES/2021/258824

MS TITLE: Direct observation of aggregate-triggered selective autophagy

AUTHORS: Anne FJ Janssen, Giel Korsten, Wilco Nijenhuis, Eugene Katrukha, and Lukas Kapitein
ARTICLE TYPE: Research Article

We have now reached a decision on the above manuscript.

To see the reviewers' reports and a copy of this decision letter, please go to: https://submit-jcs.biologists.org and click on the 'Manuscripts with Decisions' queue in the Author Area. (Corresponding author only has access to reviews.)

As you will see, the reviewers gave favourable reports but raised some critical points that will require amendments to your manuscript. I hope that you will be able to carry these out because I would like to be able to accept your paper, depending on further comments from reviewers.

We are aware that you may be experiencing disruption to the normal running of your lab that makes experimental revisions challenging. If it would be helpful, we encourage you to contact us to discuss your revision in greater detail. Please send us a point-by-point response indicating where you are able to address concerns raised (either experimentally or by changes to the text) and where you will not be able to do so within the normal timeframe of a revision. We will then provide further guidance. Please also note that we are happy to extend revision timeframes as necessary.

Please ensure that you clearly highlight all changes made in the revised manuscript. Please avoid using 'Tracked changes' in Word files as these are lost in PDF conversion.

I should be grateful if you would also provide a point-by-point response detailing how you have dealt with the points raised by the reviewers in the 'Response to Reviewers' box. Please attend to all of the reviewers' comments. If you do not agree with any of their criticisms or suggestions please explain clearly why this is so.

Reviewer 1

Advance summary and potential significance to field

© 2021. Published by The Company of Biologists under the terms of the Creative Commons Attribution License (https://creativecommons.org/licenses/by/4.0/).

The manuscript presents an elegant system to study autophagy of aggregates in mammalian cells (a relatively neglected area in terms of its morphological characteristics) and then uses the system to derive some interesting results on the sequence of events during autophagosome formation and maturation.

Comments for the author

The manuscript by Janssen et al examines a pathway of autophagy-mediated clearance of aggregated proteins caused by inducible expression of a PIM reporter fused to mKeima.
In general, how aggrephagy connects to the known spatiotemporal characteristics of the autophagic machinery has remained elusive, so a contribution towards this aim is to be welcomed.
The results are not surprising in the sense that the same sequence of events as has been described for other types of selective and non-selective autophagy appear to also be involved here, starting from the nucleation of the autophagosomal structure in the omegasome platform and continuing with incorporation of STX17 before fusion of the mature autophagosome with late endosomes/lysosomes. The timing of the whole sequence suggests that the process is efficiently executed without too much wandering about of the formed structures. It is interesting - but probably predictable - that the aggregates are rather immobile until engulfed by the autophagosome.
Overall, I found the story as presented neat and useful. An argument can be made that it is not very ambitious in the scope of the questions asked (e.g. receptors involved, role of other membranes, timing of LC3 translocation) but on balance I think the amount of work shown justifies a full publication at this point, with hopefully additional work on some of the above questions in the pipeline.
I have two criticisms:
The clearance of the PIM-mKeima reporter by autophagy should be shown to be sensitive to VPS34 inhibitions (SAR405, not wortmannin please) and/or to siRNA-mediated downregulation of elements of the ULK complex.
Please cite the work of Holzbaur et al on the spatiotemporal characteristics of mitophagy and of non selective autophagy.

Reviewer 2

Advance summary and potential significance to field

This paper from the group of Lukas C. Kapitein entitled "Direct observation of aggregate-triggered selective autophagy" does in my opinion advance the field of selective autophgay of protein aggregates (aggrephagy) by providing a tool for live cell imaging of aggrephagy. The aggregates are specifically induced by rapalog2-induced multimerization of the so-called PIM protein containing several homodimerization domains fused to mKeima. This allows live cell imaging time series studies and quantification of the events of phagophore formation, closure and acidification upon fusion of autophagosomes with lysosomes. Studies of recruitment of autophagy components and monitoring of cargo degradation during aggrephagy can now be efficiently imaged and events reled to succesful cargo degradation quantified. The timing and spatial regulation of aggrephagy is revealed importantly showing that core autophagy components and the phagophore is recruited to the aggregate instead of vice versa. This form of selective autophagy is therefore different in this aspect from bulk autophagy.
The same group has previously published this PIM-based aggrephagy analysis suytem using the EGFP-mCherry double tag. However, the use of mKeima instead of the tandem tag makes it possible to simultaneously image EGFP-tagged proteins that are expressed at low, endogenous levels.

Comments for the author

I find this study very interesting and a distinct and important step forward in cell biological studies of mechanisms of aggrephagy. The authors have used the inducible aggrepahgy model in a previous study published in Nat Commun where an EGFP-mCherry double tag was used and in the Discussion of the current paper the author have a balanced discussion of the pros and cons of the two tags as both have their strong and weak sides depending of the exact assays/applications used.
I have only a few minor issues to comment on:

© 2021. Published by The Company of Biologists under the terms of the Creative Commons Attribution License (https://creativecommons.org/licenses/by/4.0/).

In the Introduction there should be added a reference to the paper from Yoshimori's group (PMID: 18388399) also together with Jahreiss et al. 2008.
In Figure 1C it takes the reader some time (at least me) to understand the diagram. It would be better to separate the parameters in two plots to make it easier for the reader to understand.
The authors write with reference to Fig 4 and b "we observed STX17 localization to the ER and mitochondria under normal nutritional conditions (Figure 4a-b)."
However, the distribution pattern in a single image suggests this, but no markers of ER or mitochondria have been used. Although, a small side issue, such markers need to be added to validate this conclusive statement.

First revision

<u>Author response to reviewers' comments</u>

Point-by-point response

We would like to thank the reviewers for the positive and constructive feedback on our manuscript, which helped us to improve it.

Reviewer 1

1) The clearance of the PIM-mKeima reporter by autophagy should be shown to be sensitive to VPS34 inhibitions (SAR405, not wortmannin please) and/or to siRNA-mediated downregulation of elements of the ULK complex.

- In the revised manuscript we have included new data shown that clearance of the PIM-mKeima reporter is strongly reduced upon inhibition of VPS34 using SAR405. We also intended to examine clearance upon inhibition of the ULK complex using MRT67307, but the delivery of this compound has been strongly delayed. We therefore decided to proceed with the revision without these data and hope that the very clear dependence on VPS34 is sufficient evidences for macroautophagy driven mKeima-PIM clearance.

2) Please cite the work of Holzbaur et al on the spatiotemporal characteristics of mitophagy and of non-selective autophagy.

- In the revised manuscript we now cite the work of the Holzbaur lab showing the spatial and temporal regulation of autophagosome maturation along the axon (Cason et al., 2021, Maday et al, 2014 and 2012) and work showing the role of optineurin in mitophagy (Wong and Holzbaur, 2014).

Reviewer 2

1) In the Introduction there should be added a reference to the paper from Yoshimori's group (PMID: 18388399) also together with Jahreiss et al. 2008.

- In the revised manuscript, we now added the requested reference.

2) In Figure 1C it takes the reader some time (at least me) to understand the diagram. It would be better to separate the parameters in two plots to make it easier for the reader to understand.

- If we understand the comment correctly, the reviewer asks us to plot both mKeima channel values in separate graphs. However, these values by themselves are not informative as for example very big aggregates in acidic compartments can still show significant intensity in the 'pH neutral' channel, but this would be much lower than the signal in the 'pH low' channel. Therefore, only the ratio reflects the pH surrounding the mKeima, while the individual values do not. To better indicate this, we have now added a legend showing that values close to

© 2021. Published by The Company of Biologists under the terms of the Creative Commons Attribution License (https://creativecommons.org/licenses/by/4.0/).

zero correspond to "red" mKeima-PIM clusters at low pH environments (cleared), whereas values close to 1 correspond with "green" mKeima-PIM clusters at neutral pH (not yet cleared)

3) The authors write with reference to Fig 4 and b "we observed STX17 localization to the ER and mitochondria under normal nutritional conditions (Figure 4a-b)." However, the distribution pattern in a single image suggests this, but no markers of ER or mitochondria have been used. Although, a small side issue, such markers need to be added to validate this conclusive statement.

➢ We have now stained for both ER (Calnexin) and mitochondria (TOM20) in our STX17 cell line and were able to confirm that STX17 colocalized to these structures. These results are shown in Fig. S2.

Second decision letter

MS ID#: JOCES/2021/258824

MS TITLE: Direct observation of aggregate-triggered selective autophagy

AUTHORS: Anne FJ Janssen, Giel Korsten, Wilco Nijenhuis, Eugene Katrukha, and Lukas Kapitein
ARTICLE TYPE: Research Article

I am happy to tell you that your manuscript has been accepted for publication in Journal of Cell Science, pending standard ethics checks.

© 2021. Published by The Company of Biologists under the terms of the Creative Commons Attribution License (https://creativecommons.org/licenses/by/4.0/).
